# A Comparative Study of Whole Body Vibration Training and Pelvic Floor Muscle Training on Women's Stress Urinary Incontinence: Three- Month Follow- Up

**Published:** 2015-11

**Authors:** Azizeh Farzinmehr, Azar Moezy, Jalil Koohpayehzadeh, Maryam Kashanian

**Affiliations:** 1 Department of Sports Medicine, Women's General Hospital, Tehran University of Medical Sciences, Tehran, Iran; 2 Department of Sports Medicine, Iran University of Medical Sciences, Tehran, Iran; 3 Department of Community Medicine, Iran University of Medical Sciences, Tehran, Iran; 4 Department of Obstetrics and Gynecology, Iran University of Medical Sciences, Akbarabadi Teaching Hospital, Tehran, Iran

**Keywords:** Pelvic Floor Muscle Training, Stress Urinary Incontinence, Whole Body Vibration Training, RCT

## Abstract

**Objective:** To determine whether Whole Body Vibration Training (WBVT) is effective at improving pelvic floor muscles strength in women with Stress Urinary Incontinence (SUI).

**Materials and methods:** The study was designed as a randomized clinical trial. 43 women with SUI were randomly assigned in two groups; WBVT and Pelvic Floor Muscle Training (PFMT) and received interventions for four weeks. Pelvic floor muscle (PFM) strength, quality of life and incontinence intensity were evaluated. All measurements were conducted pre and post intervention and also after 3 months in all participants. The ANOVA and the independent sample t test were applied respectively to determine the differences in each group and between the groups.

**Results:** This study showed the WBVT protocol in this study was effective in pelvic floor muscles strength similar to PFMT, and also in reducing the severity of incontinence and increasing I-QOL questionnaire score. We found significant differences in each group pre and post intervention (p = 0.0001); but no significant difference in comparison of two groups' outcomes. Also after three-month follow up, there was no significant difference between groups.

**Conclusion:** The findings of this study showed the beneficial effects of WBVT in improving pelvic floor muscles strength and quality of life in patients with urinary incontinence in four-week treatment period and after three months follow up.

## Introduction

One third of adults in the community and half of the elderly in nursing homes affected by permanent or transient urinary incontinence (UI) (1). It is a distressing condition with serious implications on physical, social, psychological and sexual health of the patients (2). UI defined by the International Continence Society (ICS) as “the complaint of any involuntary leakage of urine". Stress urinary incontinence (SUI), the most common type of UI, (1-2) is the involuntary leakage of urine from the urethra with an increase in intra-abdominal pressure in the absence of detrusor muscle contraction   (3). A wide range of treatment options has been used in SUI management, including conservative interventions (pelvic floor muscle training; vaginal cones; life style interventions; anti-incontinence devices; absorbent products), pharmaceutical interventions, and surgery (4-5). 

Unfortunately, more than 50% of cases of incontinence were inadequately managed after identification (6). Pelvic Floor Muscle (PFM) dysfunction, one of the main and hidden causes in women, may lead to SUI (7). The supportive mechanism of PFM was lost with 50% of women because of childbirth and 49% of women were not able to contract the PFM in a way that increased the urethral closure pressure for urinary continence (8). PFM training is in first-line conservative management for SUI, which is intended to improve the function of the PFM (including support of the pelvic organs and contribution to the sphincteric closure mechanism of the urethra (4)); by increasing the strength, power, and also improving the timing and coordination of PFM (8). 

Whole Body Vibration Training (WBVT) is a novel intervention aimed at improving muscle strength, endurance, power, neuromuscular conditions and etc. According to previous evidences, WBVT is used to increase muscular strength due to its effects on the neuromuscular system  (9-10), muscle hypertrophy (11), improvement of pro prioceptors (12), and enlargement of slow- and fast twitch fibers and hormonal changes (10). WBVT also seems to be effective in increasing strength of weak muscles, especially in patients with different chronic diseases who are not capable of contracting their muscles (13-18). Thus the main purpose of this study was to determine whether WBVT is effective in improving PFM strength. Incontinence QOL and the severity of SUI (VAS) were also chosen as the secondary outcome measures in this study.

## Materials and methods

The ethical approval for this randomized parallel group trial was granted by the Research Ethics Committee of Iran University of Medical Sciences. It was an assessor blind study which conducted in Rasool-e-Akram Hospital. Seventy two women were primarily referred by a gynecologist after baseline evaluation. This project was supported by a grant from the Iran University of Medical Sciences (Grant Number: 12331-30-0-90). This manuscript is based on a clinical trial which was approved by the Iran University of Medical Sciences' ethical committee (No: 130/1981) and Iran Registry of Clinical Trail (IRCT ID: IRCT201012285486N1).

**Figure1:**
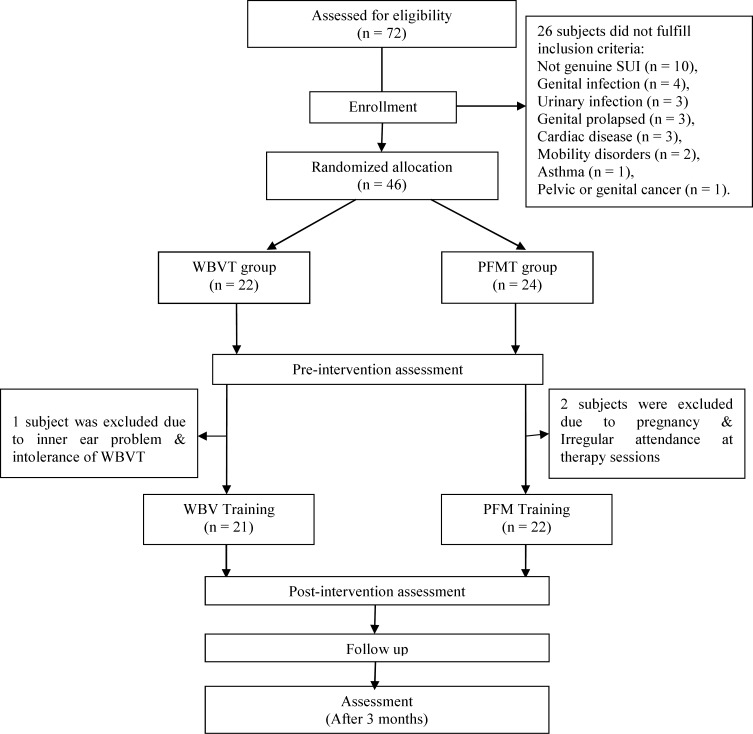
Study profile for participants in WBVT and PFMT groups

The gynecologic diagnosis was made after taking a standard history and carrying out a physical examination (inspection of the abdomen and the external genitals, vaginal exam, voiding and fluid intake diary, clinical examination with gynecological findings, urinalysis, measuring of residual urine, and cough test). Twenty six subjects did not fulfill the inclusion criteria (Figure 1). Main participants were 46 women with a history of 4.5 years proven genuine SUI. The participants aged 36-68 years, BMI = 28.6, married, mentally fit, non-pregnant, non-breast feeding , not within the 8-week postpartum period , with no genital infection, no genital prolapsed, detrusor instability, outlet obstruction, high residual urine volume and exclusion criteria included chronic constipation, pelvic or genital cancer, medical problems such as heart disease that limited activities and cardiac pacemaker, urogenital infection, any systemic disorders or drug use, neuromuscular disorders, rapid progressive pelvic prolapsus, lack of independent mobility and exercise therapy or WBVT contraindications. A list of random numbers was generated using a computer. A numbered opaque sealed envelope containing the method indicator card was opened by the secretary of the department. Women were randomly allocated to either WBVT or PFMT. Before participation, all subjects filled out the written informed consent. The gynecologist performing physical examination and a trained physical therapist who assessed PFM strength, based on Modified Oxford Grading, was blinded to group allocation and clinical data. 

The power analysis of the study was performed to detect a 10% differences PFM strength with α=0/05 and a power of 80%, a sample size of 15 per group was required, due to drop-outs , we chose to include at least 20 women in each group.

Participants were familiarized with testing procedures two days before the testing session. Before assessing PFM, all participants were educated to contract pelvic floor muscle in supine position (abducted semi-flexed hips and knees) as forcefully as possible. PFM maximal voluntary contraction (PFM strength) was assessed based on the validated, standardized modified Oxford Scale by digital palpation of a physical therapist that was trained in pelvic floor muscle evaluation. 

All participants were asked to estimate the severity of their SUI using Visual Analog Scale (VAS), a 10-cm line ranging from 0 (no incontinence) to 10 (severe incontinence) (19). 

The incontinence quality of life (I-QOL) questionnaire is used for evaluating QOL in the participants. Nojomi et al. translated and validated I-QOL questionnaire into the Persian language and approved its reliability for the Iranian population (20). It consists of 22 items evaluating concerns relating to incontinence. Subjects assigned a value on a 5 point scale from 1 (extremely) to 5 (not at all) for each item. The scores were then transformed into a zero to 100 scales. A score of 100 represented the best possible QOL and zero represented the worst.

After pre-intervention assessment, both groups began a four-week training program (three days per week) (9, 21-25). The WBVT group did their intervention based on a proposed protocol shown in table 1 on a vibration platform (Powerplate, USA) and the PFMT group was educated to do PFMT in a progressive process under supervision. PFMT consisted of static contractions of PFM associated with static contraction of hip adductors, gluteal and abdominal muscles in supine, sitting, standing, crook lying, bridging, mini-squatting, squatting positions. The PMFT group was educated to do each exercise in 3-4 set with 15-20 repetitions and a 60-second pause between set. 

During the 3-month follow-up period, the participants attended one-hour exercise classes once a month. A home-based PFMT program had been learned by patients of both group at the last session. They were encouraged to perform the home-based at least one time per day. To accurately monitor the PFMT during the follow-up period, a pamphlet illustrating the PFM strengthening exercises and a recording sheet were distributed to the subjects. The subjects were asked to document the time and sets of exercises performed at home each day. The record sheets were collected and checked once a month at the class. 

The SPSS (version 17, SPSS Inc, and Chicago, IL) was used to conduct the analysis. Normal distribution of data was determined by One-Sample Kolmogorov-Smirnov test and parametric tests were used to analyze the data. The ANOVA was applied to determine the differences in each group. An independent sample t test was used to compare the baseline measurements between the groups at the beginning and at the end of training and also to analysis change scores of both groups after the test.

**Table 1:** Characteristic of the WBVT program

**Session**	**Duration of ** **each set ** **(sec)**	**Frequency** **(Hz)**	**Amplitude** *	**Rest** **(sec)**	**Modalities**	**WBVT** **duration** **(min)**	**Sets of exercises** **								
							**a**	**b**	**c**	**d**	**e**	**f**	**g**	**h**	**i**
1	30	30	low	60	static	4	2	2	1		1			1	1
2	30	30	low	60	Static	5.5	3	3	2		1			1	1
3	30	30	low	60	Static & dynamic	6.5	3	3	3		1			1	2
4	30	35	low	50	Static & dynamic	8	3	3	3	1	2		1	1	2
5	45	35	low	50	Static & dynamic	12	2	2	3	2	2		1	2	2
6	45	35	low	50	Static & dynamic	12	2	2	3	2	2		1	2	2
7	45	40	high	40	Static & dynamic	13.5	2	2	3	2	2	1	2	2	2
8	45	40	high	40	Static & dynamic	15	2	2	3	2	3	1	2	2	3
9	45	40	high	40	Static & dynamic	15	2	2	3	2	3	1	2	2	3
10	60	40	high	30	Static & dynamic	16	1	1	2	2	2	2	2	2	2
11	60	50	high	30	Static & dynamic	16	1	1	2	2	2	2	2	2	2
12	60	50	high	30	Static & dynamic	16	1	1	2	2	2	2	2	2	2

a: Static position, sitting with hip joints abduction and feet out of platform; b: Static position , sitting with hip joints adduction and feet out of platform; c: Static position, sitting with hip joints abduction and feet out of platform while patient tries to contract her hip adductors statically; d: Static position, sitting cross-legged; e: Static position, sitting cross-legged while patient tries to press her buttock to vibration platform.; f: Dynamic position, sitting with hip joints abduction and feet out of platform while patient tries to bend successively hip joints (first right and then left hip); g: Dynamic position, sitting with hip joints adduction and feet out of platform while patients try to pull her knees into the abdomen; h: Dynamic position, sitting with hip joints adduction and feet out of platform, while patient keeps her arms crossed on the chest and bends her trunk forward and backward; i: Dynamic position, sitting with hip joints adduction and feet out of platform, while patient keeps her arms crossed on the chest and bends her trunk to the sides.

* Amplitude: High = 5 mm, Low = 2.5 mm;

** Exercises:

The change scores of a group were defined as the increase or decrease of each variable from pre-test to post-test. The level of significance was set at p ≤ 0.05. In order to study the improvement process of participants, the progression rate of all data were also calculated as following equation.


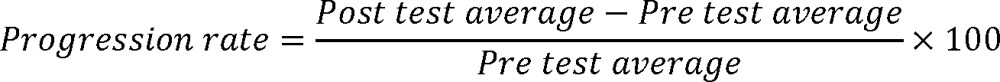


To assess the intra-tester reliability of objective tests, ten women had repeated measurements seven days apart in a pilot study. Test-retest reliability of pelvic floor muscle strength by Modified Oxford Grading System was assessed using Kendall exam (p ≤ 0.0001). The correlation between the first and second pelvic floor muscle strength was r = 0.857 and p value = 0.003.

## Results

There were no significant differences between WBVT and PFMT groups for the demographic variables, indicating that the groups were well matched. The rate of postmenopausal women in PFMT group was 82%and in WBVT group was 88%. During the second and third weeks of training, three subjects withdrew (Figure 1). All remaining patients completed four weeks of training and the evaluation sessions. In both groups, subjects acquainted very rapidly with the training protocols. There were no reports of side effects, dissatisfaction or discomfort in both groups. All subjects of WBVT group, about their experience of receiving treatment, stated that the vibration training was enjoyable but a fatiguing modality. 

Independent- sample t test revealed no significant differences (p ≤ 0.05) between two groups at the beginning of the study, but there were significant differences in all variables in the WBVT group and PFMT group (Table 2) between pre and posttests (p ≤ 0.05).

There were not significant differences in the changing score of all data between two groups (Table 3).No statistically significant differences were found in the changing scores of all data between 36 subjects of PFMT (n = 19) and WBVT (n = 17) after 3 month follow up which means the therapeutic effects of both protocols were similar (Table 4).

**Table 2:** Training Effect within Groups

	**Tests**	**Pre test value**	**Post test value**	**3 months value**	**P value** **(p ≤ 0.05)**
		**Mean ± SD**	**Mean ± SD**	**Mean ± SD**	
WBVT group (n = 21)	I-QOL^1^	43.52 ± 26.64	64.02 ± 26.5	63.38 + 26.41	0.0001*
	ALB ^2^	40.33 ± 23.68	59.02 ± 27.35	59.32 + 25.75	0.0001*
	PS^3^	51 ± 31.9	71.68 ± 26.34	70.89 + 26.93	0.0001*
	SE^4^	34.09 ± 30.53	58.33 ± 30.6	56.47 + 31.9	0.0001*
	PFM strength^5^	2.45 ± 0.9	4.10 ± 0.7	3.94 + 0.89	0.0001*
	VAS _(0-10)_^6^	7.95 ± 1.9	4.14 ± 1.9	3.53 + 1.87	0.0001*
PFMT group (n = 22)	I-QOL	56.66 + 22.57	74.47 + 16.71	75.21 + 19.04	0.0001*
	ALB	50.38 + 21.11	64.64 + 17.61	71.21 + 19.22	0.0001*
	PS	67.92 + 24.92	83.95 + 17.84	83.03 + 18.21	0.0001*
	SE	46.45 + 29.61	69.31 + 23.1	67.63 + 26.94	0.0001*
	PFM strength	2.66 + 0.96	4.18 + 0.85	4.26 + 0.80	0.0001*
	VAS _(0-10)_	7.42 + 1.64	3.05 + 1.46	3.11 + 2.40	0.0001*

1-I-QOL: Incontinence quality of life; 2-ALB: Avoidence and limiting behavior; 3-PS: Psychosocial impacts; 4-SE: Social embarrassment; 5-PFM Strength: Pelvic Floor Muscle Strength; 6-VAS: Visual analog scale

* Significant difference between pre test and post test values, p ≤ 0.05

**Table 3:** Comparison between groups

**Tests**	**WBVT group [(n = 21)]**	**PFMT group [(n = 22)]**	**P value** **(p ≤ 0.05)**
	**Changing score** **Mean ± SD**	**Changing score** **Mean ± SD**	
I-QOL	21.72 ± 17.3	18.75 ± 13.9	0.538
ALB	19.44 ± 20.6	14.92 ± 14.5	0.411
PS	21.94 ± 18.1	17.68 ± 13.1	0.382
SE	26.1 ± 19.8	22.95 ± 23.9	0.633
Strength	1.57 ± 0.8	1.54 ± 0.5	0.905
VAS _(0-10)_	-3.9 ± 2.02	-4.36 ± 2.01	0.460

**Table4:** Comparison between training groups after 3 month follow up

	**WBVT group [(n = 17)**]	**PFMT group [(n = 19)**]	**P value** **(p ≤ 0.05)**
	**Changing score** **Mean ± SD**	**Changing score** **Mean ± SD**	
IQOL	18.68 ± 16.94	23.07 ± 14.98	0.852
ALB	16.68 ± 22.83	25.16 ± 16.32	0.216
PS	19.43 ± 18.78	20.62 ± 16.85	0.477
SE	22.05 ± 18.20	24.21 ± 23.28	0.200
Strength	1.52 ± 1.06	1.68 ± 0.58	0.025
VAS _(0-10)_	-4.29 ± 2.71	-4.26 ± 2.49	0.530

## Discussion

The effect of WBV on strength of extermities' muscles have been demonstrated in various studies (9, 21-25). Thus; in the present study, wedesigned a four-week protocol to investigate the short term effect of WBVT on PFM strength and also on SUI severity and I-QOL in incontinent women.

The results of our study indicated that after a short term interventions, the improvement in the PFM tone and strength was occurred in both groups which may be due to increasing PFM strength and function within PFMT and the co-contraction of pelvic floor, hip, abdominal and other synergists during WBVT. Improvement of PFM power during contraction are essential in the conditions that intra-abdominal pressure is suddenly increased. It seems WBV could have the potential to activate PFM and improve their function.The change in pelvic floor muscle function is not the only effect of WBVT. It is also possible that other aspects of muscle activity that were not measured in this trial, such as timing,coordination, endurance and rapidity of contraction,might contribute to the perception of improvement in SUI. Cronin et al. indicated that muscle stiffness and intramuscular temperature were enhanced due to WBVT. Both of these factors could have great impacts in muscle strength increase (26). In addition to the effects of WBVT on muscle strengthening (24), WBVT is a somatosensory stimulus for proprioception with a long-lasting postural effect , Ia and II afferent fibers of PFM muscle spindles are sensitive to small alteration of muscle length stimulated by vibration, stimulation of proprioceptive receptors could initiate stretch and cutaneous reflexes (12). Also the improvement in PMF function in WBVT group might also due to its effects on the neuromuscular system (high firing rate in high threshold muscle units, tonic vibration reflex, synchronization of muscle units)  (9-10), muscle tuning (21), muscle hypertrophy (11), improvement of proprioceptors (12) and massive stimulation of propriospinal reflex pathways (10).

Although there were no significant differences between our two groups at baseline, in WBVT group the mean of progression rate of PFM strength was 90.87 % while it was 76.13 % in PFMT group; it seems that WBVT may be an effective approach as a multi-muscles strengthening modality in SUI management. Based on the patients' feedback in WBVT group, they felt quick recovery in the PFM strength; perhaps it may be to the activation of all groups of the PFM, trunk and lower limb muscles.

Several studies have shown up to remarkable improvement in symptoms of all age groups SUI following PFMT (2, 3, 27, 28). The results of our study were determined women with SUI do better in an exercise protocol carefully supervised by a specialist similar to the results of Zanetti et al. (29) The reasons for this may be that if the woman fully understands how she can help herself and if she has had adequate time to explain her problem with the specialist, she will have a better compliance with exercise regimes and the result of exercise therapy will be more successful. 

Our results revealed a significant difference in PFM strength and reduction of SUI symptoms between pre and post test results in PFMT group and the progression rate for PFM strength was 76% that may be due to strengthening and co-contraction pelvic floor muscles. Moreover, increasing muscle strength may often enhance stiffness of the PFM, although this parameter has not been measured in this study.

The therapeutic effects of our approach in the PFMT group seemed to be similar to that of PFMT when compared with previous studies. Kim et al. demonstrated that multi-dimensional exercise therapy was also effective in reducing SUI (27). 

In the present study, VAS was used as a subjective evaluation for the patient to clarify the severity of their incontinence. Abdulaziz et al. also used a five point VAS for subjective assessment of SUI and showed an improvement in VAS results after exercise therapy (30). VAS was also used by Stach-Lempinen et al. to assess the subjective improvement of incontinence symptoms (19). Similar to the results of previous studies, our results also revealed a significant difference in VAS between pre and post interventions in both groups, but there was no significant difference between PFMT and WBVT groups . It seems both interventions used in this study were effective in decreasing SUI severity.

This clinical trial was also designed to investigate the short term effects of WBVT and PFMT on the QOLin our participants after 4 weeks. The total score of I-QOL and sub -scores [Avoidance and Limiting Behavior, Psychosocial Impacts, Social Embarrassment] in both WBVT and PFMT groups showed significant differences between pre and post interventions, similar to the previous studies (19, 28, 31). 

Although there was not a significant difference between I-QOL findings of these two groups, the percent of increasing I-QOL total score was 84.8 % in WBVT group and 60% in PFMT which revealed the acceptance of WBVT approach as an effective method in management of SUI and improve their quality of life. Based on the results of the current study, the social embarrassment sub- score has increased dramatically relative to the other sub-scores which represent a great improvement in the ability of patients to participate in social activities.

The results of our follow-up study showed the therapeutic effects were still remained after 3 months and the patients were satisfied with their treatment which is one of the strengths of this research program. 

The findings of the present study have indicated the significant effects of WBVT as well as PFMT in increasing PFM strength, improving the scores of the I-QOL and in reducing incontinence severity, improve awareness of patients regarding PFM and the perineal area, reducing patients' stress, improving their mood and promotion of social life of both groups. The main limitations of this study are the lack of a control group (no treatment) due to ethical issues and also the assessment of SUI was self-reported and not based onurodynamic examination. Another limitation of this study is the lack of prior WBVT protocols; therefore our protocol for WBVT may not have been optimal.

It seems that WBVT has had equal effects with PFMT as an effective method in increasing PFM strength of women with SUI and improved patients' physical and psychosocial health. The positive effects of WBVT, especially its short period of training and its remaining effects for 3 months, support the need for further study to evaluate the efficacy of this treatment.
